# Screening and routine diagnosis of mental disorders among migrants in primary care: A cross-sectional study

**DOI:** 10.1016/j.jmh.2023.100205

**Published:** 2023-11-10

**Authors:** Stella Evangelidou, Angeline Cruz, Yolanda Osorio, Ethel Sequeira-Aymar, Alessandra Queiroga Gonçalves, Laura Camps-Vila, Marta M. Monclús-González, Alba Cuxart-Graell, Elisa M. Revuelta-Muñoz, Núria Busquet-Solé, Susana Sarriegui-Domínguez, Aina Casellas, M. Rosa Dalmau Llorca, Carina Aguilar Martín, Ana Requena-Mendez

**Affiliations:** a Barcelona Institute for Global Health (ISGlobal, Hospital Clínic-Universitat de Barcelona). Carrer Roselló 132, 40, 08036 Barcelona, Spain; b Parc Sanitari Sant Joan de Deu, Programa Atenció a la Salut Mental de les persones Immigrades i Programa per Persones Sense Llar, Carrer Dr. Antoni Pujadas, 42, 08830 Barcelona, Spain; c Consorci d'Atenció Primària de Salut Barcelona Esquerra (CAPSBE) Casanova, Carrer Rosselló 161, 08036 Barcelona, Spain; d August Pi i Sunyer Biomedical Research Institute (IDIBAPS), Carrer Rosselló, 149, Barcelona, Spain; e Unitat de Suport a la Recerca Terres de l'Ebre, Fundació Institut Universitari per a la recerca a l'Atenció Primària de Salut Jordi Gol i Gurina (IDIAPJGol), 43500 Tortosa, Tarragona, Spain; f Red de Investigación en Cronicidad, Atención Primaria y Promoción de la Salud (RICAPPS), Spain; g Unitat Docent Multiprofessional d'Atenció Familiar i Comunitària Catalunya Central, Institut Català de la Salut, Carrer Pica d'Estats, 13-15, 08272 Sant Fruitós de Bages, Barcelona, Spain; h Centre d'Atencio Primaria Sagrada Família, Consorci Sanitari Integral (CSI), Carrer Còrsega 643, 08025 Barcelona, Spain; i Centre d'Atencio Primaria Rambla Ferran, Institut Català de la Salut (ICS), Carrer Rambla Ferran 44, 25007, Lleida, Spain; j Centre d'Atencio Primaria Sagrada Família, Institut Català de la Salut, Carrer St. Cristòfol, 34, 08243 Manresa, Barcelona, Spain; k Centre d'Atencio Primaria 1 Maig, Institut Català de la Salut (ICS), Carrer De la Mercè, 5, 25003, Lleida, Spain; l Equip d'Atenció Primària Tortosa Oest, Institut Català de la Salut, 43500, Tortosa, Tarragona, Spain; m Unitat d'Avaluació, Direcció d'Atenció Primària Terres de l'Ebre, Institut Català de la Salut, Tortosa, 43500 Tarragona, Spain; n Department of Medicine Solna, Karolinska Institutet, Solnavägen 17177, Solna, Stockholm, Sweden; o CIBERINFEC, ISCIII-CIBER de Enfermedades Infecciosas, Instituto de Salud Carlos III, Centro de Investigación Biomédica en Red de Enfermedades Infecciosas, Madrid, Spain

**Keywords:** Mental health, Migrant, Screening, Disorders, Primary care, Electronic health records

## Abstract

**Background:**

Migrants in host countries are at risk for the development of mental health conditions. The two aims of the study were to describe routine diagnoses of mental disorders among migrant patients at primary healthcare level and the associated risk factors, and to test the utility of an innovative migrant mental health assessment by evaluating whether the health professionals followed the recommendations proposed by the clinical decision support system (CDSS) tool.

**Methods:**

A cross-sectional study was carried out in eight primary care centres (PCCs) in four non-randomly selected health regions of Catalonia, Spain from March to December 2018. Routine health data and mental health diagnoses based on the International Classification of Diseases (10th edition), including mental, behavioural and neuro developmental disorders (F01-F99), symptoms and signs involving emotional state (R45), and sleep disorders (G47), were extracted from the electronic health records. The proportion of mental health conditions was estimated and logistic regression models were used to assess any possible association with mental health disorders. The utility of the mental health assessment was assessed with the proportion of questionnaires performed by health professionals for migrants fulfilling the mental health screening criterion (country of origin with an active conflict in 2017) and the diagnoses given to the screened patients.

**Results:**

Of 14,130 migrants that visited any of the PCCs during the study period, 7,358 (52.1 %) were women with a median age of 38.0 years-old. There were 520/14,130 (3.7 %) migrant patients diagnosed with a mental disorder, being more frequent among women (342/7,358; 4.7 %, *p*-value < 0.001), migrants from Latin-America (177/3,483; 5.1 %, *p* < 0.001) and those who recently arrived in Spain (170/3,672; 4.6 %, *p* < 0.001). A lower proportion of mental disorders were reported in migrants coming from conflicted countries in 2017 (116/3,669, 3.2 %, *p* = 0.053).

Out of the 547 mental health diagnoses reported in 520 patients, 69/14,130 (0.5 %) were mood disorders, 346/14,130 (2.5 %) anxiety disorders and 127/14,130 (0.9 %) sleeping disorders. Mood disorders were more common in migrants from Eastern Europe (25/2,971; 0.8 %, *p* < 0.001) and anxiety disorders in migrants from Latin-America (126/3,483; 3.6 %, *p* < 0.001), while both type of disorders were more often reported in women (*p* < 0.001).

In the adjusted model, women (aOR: 1.5, [95 % CI 1.2–1.8, *p* < 0.001]), migrants with more than one visit to the health center during the study period (aOR: 4.4, [95 %CI 2.8–6.8, *p* < 0.001]) and who presented an infectious disease (aOR: 2.1, [95 %CI 1.5–3.1, *p* < 0.001]) had higher odds of having a mental disorder.

Lastly, out of the 1,840 migrants coming from a conflicted country in 2017 who were attended in centres where the CDSS tool was implemented, 29 (1.6 %) had a mental health assessment performed and the tool correctly identified one individual.

**Conclusions:**

Mental health is a condition that may be overlooked in migrants at primary healthcare. Interventions at this level of care must be reinforced and adapted to the needs and circumstances of migrants to ensure equity in health services.

## Highlights

• Mental disorders represent a high burden in migrants, especially women, newly arrived migrants and frequent visitors of primary care facilities.
• Anxiety, sleep and mood disorders are the most common conditions diagnosed among migrants at primary care on a routine basis.
• Migrant mental health may be overlooked at primary care.
• Adequate innovative mental health screening assessments targeting the migrant patients must be developed and integrated into electronic health records.

## Introduction

Migration is a global and increasing phenomenon with 281 million international migrants in 2020 (Aksoy and Poutvaara, 2019; International Organization for Migration 2022). As of 2020, 15.2 % of the Spanish population was foreign-born, including 10.6 % born in a non-European country, largely from Latin America and Eastern Europe (International Organization for Migration 2022; Instituto Nacional de Estadística 2021). Immigration numbers rank Spain among the most popular destinations for international migrants, being the fourth country in Europe and the 10th worldwide (The World Bank 2022; McAuliffe and Triandafyllidou, 2021).

Migrant populations of host countries are at higher risk for the development of mental health conditions compared with the autochthonous population (Bedaso and Duko, 2022), particularly in people who have been exposed to war, armed conflict, political instability, and other types of organized violence (Mesa-Vieira et al., 2022), such as trafficking (Goldenberg, 2015). Pre-migration trauma does predict mental disorders, but the post-migration context can be an equally important determinant of mental health (Hynie, 2018). Accordingly, post-migration living difficulties were reported to be more strongly associated with psychological distress than the socio-demographic characteristics of the individuals (Schick et al., 2016). The risk of certain mental disorders, such as psychosis, increases among migrants after their arrival at the host country (Termorshuizen and Selten, 2022), whereas other mental disorders, such as anxiety and affective disorders, may be present during pre-departure and transit migration stages (Hynie, 2018). Conversely, several studies observed that soon after arriving in their host country, migrants typically demonstrate lower rates of common mental health problems compared with native-born, corroborating the “healthy immigrant effect” (Calderón-Larrañaga et al., 2011; Constant et al., 2018). However, over time, the rate increases and becomes like in native-born populations (Kirmayer et al., 2011; Kennedy et al., 2015; McDonald and Kennedy, 2004).

General practitioners (GPs) are the healthcare professionals that most commonly attend migrants with mental health needs. Compared to the general population, migrants are less likely to seek out care for mental health conditions (Lu et al., 2020). Each year, approximately 10–20 % of migrants consult a GP for a mental health problem (Aery and Mckenzie, 2018). Although several studies have estimated the burden of mental disorders among migrant populations at host countries (Greenhalgh, 2009), to our knowledge no studies have focused on the routine diagnosis of mental disorders at primary care level among migrants in Spain. There exist various assessment tools to detect and diagnose mental disorders. However, these tools are complex, contain an excessive number of questions, and require a significant amount of time to administer, leading to low participation and completion rates. Additionally, the results obtained from these tools must be analysed and interpreted manually by health professionals, which may yield inaccurate diagnoses. Clinical decision support systems (CDSS) have been implemented in primary care to support practitioners in clinical decision making (Røst et al., 2020). Computerized-CDSS can reduce inconsistencies in the identification, assessment and management of mental health problems by GPs by guiding them through the consultation (Horrocks et al., 2018).

We hypothesized that mental health needs among migrants are not properly addressed at primary care in Spain. Our study aimed i) to describe the common mental disorders diagnosed on routine basis among migrants at primary care level in Catalonia, Spain, while exploring the associated risk factors towards the development of such conditions and ii) to test the utility of a migrant mental health screening strategy considering whether the health professionals followed the screening recommendations supported by the computerized-CDSS.

## Methodology

### Study design and population

This is a cross-sectional study carried out in eight primary care centres (PCCs) located in four areas of Catalonia, Spain: Barcelona, Manresa, Lleida, and Tortosa, from March to December of 2018. All areas have a high migrant density accounting for 20 % or more of the total population (Instituto de Estadística de Catalunya, 2019). This study was part of a pilot clustered-randomized-controlled trial where an opportunistic standardized screening program was implemented in eight PCCs based on an individualized risk assessment for each migrant (Sequeira-Aymar et al., 2021). As part of the screening strategy, a CDSS implemented into the electronic health record (EHR) system of four randomly allocated PCCs sent real-time prompts to health professionals for the screening of different conditions targeting migrant patients. The screening program included seven infectious diseases, female genital mutilation, and a mental health assessment. To evaluate the effectiveness of the intervention, the four PCCs with the CDSS integrated into the EHR system were compared to the four centres which followed the standard of care (Sequeira-Aymar et al., 2021)

The study population included all migrants ≥15 years-old, who visited any of the eight PCCs during the intervention period for any reason and were born in the areas of Eastern Europe (EE), Latin America (LA), Northern Africa (NA), Sub-Saharan Africa (SSA), Middle East, Eastern and Southeast Asia, using convenient sampling methods. Migrant patients coming from countries of Northern and Southern Europe, Anglo-Saxon America, and Oceania, categorized under Western countries due to sociocultural, political, and economic criteria (Shvili, 2021), were excluded from the study (Annex 1).

### Study procedures

Firstly, face-to-face training sessions on migrant mental health were conducted for the healthcare staff, including nurses and GPs at all eight PCCs of the study (a one-hour session per center). The trainer was a psychiatrist expert in migrant mental health. The sessions covered background information on intercultural competence, common mental disorders in migrants included in the target population, screening and diagnostic processes, treatment recommendations and care pathway referral criteria to specialist care.

During the intervention, health professionals from the four intervention centres, received prompts for a mental health screening assessment. The assessment was recommended to health professionals for migrant patients coming from countries in conflict in 2017 as reported by the institution Escola Cultura de Paz (2018) (Escola de Cultura de Pau, 2018). This screening criterion was agreed in a consensus workshop with experts on migrant mental health and GPs, and has been published elsewhere (Sequeira-Aymar et al., 2020). When the screening recommendation was taken into consideration, GPs had to fill in a short screening questionnaire of seven dichotomous questions which included: year of arrival in Spain, experience/witnessing episode(s) of violence during migration trajectory, substance abuse, adjustment disorders, sleeping disorders, difficulty falling sleep, disruptive sleep disorders and insomnia. The questions were based on general clinical observations at specialized migrant mental health units.

After the screening assessment was completed, it was the responsibility of the GPs, as part of the standard procedures of each PCC, to complete the follow-up and refer the patient to a mental health specialist when required.

### Data extraction

Routine health data were retrospectively and pseudo-anonymously collected from the SIDIAP (Sistema d'Informació per al Desenvolupament de la Investigació en Atenció-Primària) database (Recalde et al., 2022). For the purpose of this study the data collected included mental health diagnoses based on the International Classification of Diseases tenth revision (ICD-10). Mental, behavioural and neurodevelopmental disorders codes (F01-F99), symptoms and signs involving emotional state (R45), and sleep disorders (G47) were included in the analysis. Additional information extracted was socio-demographic data (country of birth, age, and sex), entry and exit date to the PCCs, number of visits during the intervention period, whether the patient came from an area in conflict in 2017, a laboratory test or the diagnosis of the seven infectious diseases included in the screening program [human immunodeficiency virus (HIV), active tuberculosis, viral hepatitis B and C, strongyloidiasis, schistosomiasis, Chagas] to assess the comorbidity with an infectious disease, and the dichotomous variables collected in the short screening questionnaire on mental health.

For each PCC, we included the health region and the Mortality in small Spanish areas and Socioeconomic and Environmental Inequalities (MEDEA) index, a socioeconomic deprivation score estimated in health settings in Spain (Domínguez-Berjón et al., 2008). It is a useful instrument for the detection of unfavorable socioeconomic characteristics, related to work, education and housing conditions, in specific areas of large cities in Spain (Garcia-Gil et al., 2014). This deprivation index is classified into quintiles, from Medea 1 (low deprivation) to Medea 5 (high deprivation) and it is a numerical continuous variable depending on the demographic census data. In this sense, there are minimum and maximum scores. Duque et al. (Duque et al., 2021) have estimated MEDEA scores in Spain into five quintiles that range from −2.58 to 4.88 [Medea 1 (low): −2.58/−0.86; Medea 2 (intermediate low): −0.87/−0.27; Medea 3 (intermediate): −0.28/−0.21; Medea 4 (intermediate high): 0.22/0.82; Medea 5 (high): 0.83–4.88].

Country of birth was extracted and aggregated into geographical areas of birth adapting the international classification of GeoSentinel (Annex 1) (Field et al., 2010). Age was categorized as <18 years (minors), 18–35 years (early adulthood), 36–55 years (mid adulthood), and >55 years (late adulthood). Finally, the time registered in the Catalan health system was categorized based on the European center for Disease Prevention and Control definition of newly arrived migrants (<5 years) and long-term residing migrants (≥5 years) (European Centre for Disease Prevention and Control, 2018).

### Statistical analysis

The primary outcome measure was the diagnosis of any mental disorder based on ICD-10 codes registered by GPs in migrant patients at any of the eight PCCs.

Summary statistics were presented as frequencies for categorical variables and as means with the standard deviation (SD) for normally distributed continuous variables or medians with interquartile range (IQR) for non-normally distributed continuous variables. Associations were tested with Fisher's exact or Chi-square tests for categorical variables. For normally distributed quantitative variables, *t*-tests, or one-way analysis of variance (ANOVA) were performed, while for not normally distributed quantitative variables, Wilcoxon Rank-Sum and Kruskal-Wallis tests were carried out. Logistic regression models were used to identify associations between the outcome, presenting a mental disorder, and the exposure variables, which included socio-demographic characteristics (area of birth, age and sex), coming from an area with an active conflict in 2017, variables related to health services (time registered in the Catalan health service, visits to the PCC, being attended at the intervention centres (where the CDSS tool was implemented in the EHR system for the mental health assessment), and the health region where the PCC is located) and presenting any of the infectious diseases included in the screening program. Odds ratio (OR), adjusted odds ratio (aOR) and 95 % confidence interval (CI) were computed.

For the utility of the mental health assessment tool, we assessed the proportion of questionnaires performed by health professionals, following the screening prompt made by the CDSS, and the diagnoses given to the assessed migrant patients.

The significance level was established at the 5 % level and variables with missing values higher than 10 % were not considered for the analysis of the study. Stata-IC-16.0 was used as the statistical software (Stata-16. TX:Stata-Corp).

### Ethical considerations

This study was approved by the Ethics committee of Hospital-Clínic, Barcelona (HCB/2016/0858) and the Fundació Institut Universitari per a la recerca a l'Atenció Primària de Salut Jordi Gol i Gurina (IDIAPJGol) (4R17/066). The study was reported using the STROBE (Strengthening the Reporting of Observational studies in Epidemiology) guidelines (Annex 2).

## Results

### Socio-demographic characteristics of the study population

**Fig. 1:**
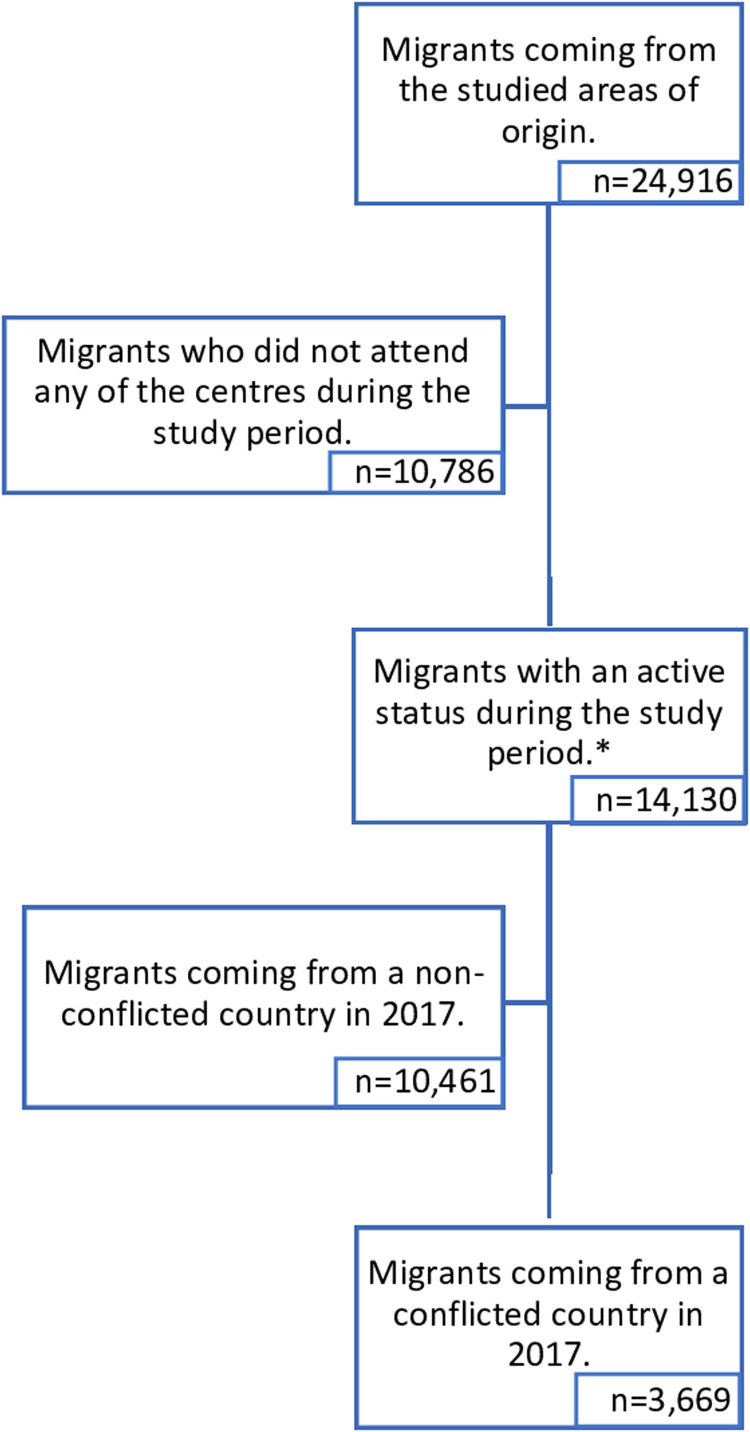
Flowchart of the study population *Study population.

The total number of migrants with any record registered in the eight PCCs was 24,916 and 14,130 (58.4 %) of them attended any of the eight participating centres at least once from March to December in 2018 (Fig. 1).

Table 1 shows the characteristics of the study population. The median age was 38.0 (IQR: 30.0–47.0) years old. Half of the migrant cohort were women (7358/14,130, 52.4 %). Overall, patients came more frequently from NA (4587/14,130, 32.5 %) and LA (3483/14,130, 24.7 %) countries (Table 1). Further, 3669/14,130 patients (26.0 %) came from a country of birth where an active conflict took place in 2017, with a high proportion of individuals in the PCCs of Barcelona (1022/2389, 42.8 %). The mean number of visits of all migrants was 4.0 (IQR: 2.0–7.0), meaning that the vast majority visited the PCCs more than one time (11,937/14,130, 84.5 %). A high proportion of patients were registered in the Catalan health system for ≥5years (10,458/14,130, 74.0 %). However, in the health region of Barcelona the vast majority were, considered newly arrived migrants (registered <5years) (2354/2389, 98.5 %) (Table 1). According to the MEDEA index, the lowest socioeconomic deprivation rate was attributed to the PPCs in Manresa (0.4, SD 0.33) and in Lleida (0.6, SD 0.01).

**Table 1:** General characteristics of the study population.

	Barcelona	Lleida	Tortosa	Manresa	Total
	n (%)	n (%)	n (%)	n (%)	n (%)
**Total**	2389	5158	3419	3164	14,130
Age (in years)*	37.0 (30.0–46.0)	40.0 (32.0–48.0)	39.0 (29.0–47.0)	38.0 (29.0–46.0)	38.0 (30.0–47.0)
**Age category**					
<18 years 18–35 years 36–55 years >55 years	54 (2.3) 1033 (43.2) 1020 (42.7) 282 (11.8)	152 (3.0) 1715 (33.3) 2782 (53.9) 509 (9.9)	131 (3.8) 1247 (36.5) 1705 (49.9) 336 (9.8)	114 (3.6) 1246 (39.4) 1510 (47.7) 294 (9.3)	451 (3.2) 5241 (37.1) 7017 (49.7) 1421 (10.1)
**Sex**					
Women Men	1515 (63.4) 874 (36.6)	2420 (46.9) 2738 (53.1)	1671 (48.9) 1748 (51.1)	1752 (55.4) 1412 (44.6)	7358 (52.1) 6772 (47.9)
**Area of birth**+					
EE LA NA SSA Southern Asia and Middle East Eastern and Southeast Asia	265 (11.1) 1399 (58.6) 149 (6.2) 28 (1.2) 134 (5.6) 414 (17.3)	1318 (25.6) 859 (16.7) 1530 (29.7) 1275 (24.7) 176 (3.4) 0 (0.0)	849 (24.8) 486 (14.2) 1439 (42.1) 172 (5.0) 473 (13.8) 0 (0.0)	539 (17.0) 739 (23.4) 1469 (46.4) 314 (9.9) 103 (3.3) 0 (0.0)	2971 (21.0) 3483 (24.7) 4587 (32.5) 1789 (12.7) 886 (6.3) 414 (2.9)
**Country of birth in conflict in 2017**	1022 (42.8)	1411 (27.4)	799 (23.4)	437 (13.8)	3699 (26.0)
Number of visits*	4.0 (2.0–6.0)	4.0 (2.0–8.0)	4.0 (2.0–7.0)	4.0 (2.0–7.0)	4.0 (2.0–7.0)
**Visits during the study period**					
1 visit >1 visit	417 (17.5) 1972 (82.5)	764 (14.8) 4394 (85.2)	583 (17.1) 2836 (82.9)	429 (13.6) 2735 (86.4)	2193 (15.5) 11,937 (84.5)
**Time registered in the system**					
<5 years ≥5 years	2354 (98.5) 35 (1.5)	632 (12.3) 4526 (87.8)	290 (8.4) 3129 (91.5)	396 (12.5) 2678 (87.5)	3672 (26.0) 10,458 (74.0)
**MEDEA index**⁎⁎	0.9 (0.12)	0.6 (0.01)	0.9 (0.05)	0.4 (0.33)	0.7 (0.28)
**Individuals diagnosed with at least one mental disorder**	114 (4.8)	198 (3.8)	85 (2.5)	123 (3.9)	520 (3.7)
**Individuals diagnosed >1 mental disorder**	9 (0.4)	15 (0.3)	6 (0.2)	6 (0.2)	36 (0.3)
**Individual diagnosed with at least one ID**^	121 (5.1)	247 (4.8)	58 (1.7)	56 (1.8)	482 (3.4)
**Individuals with a mental disorder and an ID**^	7 (0.3)	18 (0.4)	2 (0.1)	7 (0.2)	34 (0.2)
**Migrants with a questionnaire answered as part of the screening strategy**	0 (0.0)	12 (0.2)	0 (0.0)	17 (0.5)	29 (0.2)

+ See Annex 1 for details of the classification of countries in areas of birth: Eastern Europe (EE), Latin-America (LA), Northern Africa (NA), and Sub-Saharan Africa (SSA); Infectious diseases (ID).

^ Infectious diseases included in the study: Human immunodeficiency virus (HIV), Viral hepatitis B and C, Active Tuberculosis, Chagas disease, Strongyloidiasis and Schistosomiasis.

⁎ Presented in median and IQR.

⁎⁎ Presented in mean and SD.

### Mental disorders

There were 520/14,130 (3.7 %) migrants diagnosed with at least one mental disorder during a routine consultation with a GP (Table 1). In addition, 36/14,130 (0.3 %) had more than one type of mental health diagnoses and 34/14,130 (0.2 %) were also diagnosed with one of the infectious diseases included in the screening program (Table 1).

Overall mental disorders were most frequent among women (342/7358; 4.7 %, p-value<0.001) and among LAs (177/3483, 5.1 %), followed by EEs (139/2971, 4.7 %), NAs (137/4587, 3.0 %), and SSAs (43/1789, 2.4 %) (*p* < 0.001) (Table 2). Further, mental disorders were less frequent in migrants registered in the Catalan health system for ≥5 years (350/10,458; 3.4 %, *p* < 0.001) and among migrants coming from a country in conflict during 2017 (116/3669; 3.2 %, *p* = 0.053), of which 67 were long residing and 49 newly arrived migrants.

**Table 2:** Factors associated with mental health disorders.

	Bivariate associations		Simple Firth Logistic Regression	Adjusted Firth Logistic Regression	
	Total n/N (%)	*p*-value*	OR (95 %CI)	aOR (95 %CI)	*p*-value
**Total**	120/				
**Age category**					
<18 years 18–35 years 36–55 years >55 years	12/451 (2.7) 191/5241 (3.6) 269/7017 (3.8) 48/1421 (3.4)	0.537	reference 1.4 (0.8–2.5) 1.5 (0.8–2.6) 1.3 (0.7–2.4)	reference 1.2 (0.7–2.2) 1.3 (0.7–2.4) 1.0 (0.5–2.0)	0.403
**Sex**					
Women	342/7358 (4.7)	**<0.001**	1.8 (1.5–2.2)	1.5 (1.2–1.8)	**<0.001**
**Area of birth**+					
EE LA NA SSA Southern Asia and Middle East Eastern and Southeast Asia	139/2971 (4.7) 177/3483 (5.1) 137/4587 (3.0) 43/1789 (2.4) 16/886 (1.8) 8/414 (1.9)	**<0.001**	reference 1.1 (0.9–1.4) 0.6 (0.5–0.8) 0.5 (0.4–0.8) 0.4 (0.2–0.6) 0.4 (0.2–0.8)	reference 1.0 (0.8–1.3) 0.7 (0.5–0.9) 0.5 (0.3–0.7) 0.5 (0.3–0.8) 0.3 (0.2–0.7)	**<0.001**
**Country of birth in conflict in 2017**	116/3669 (3.2)	**0.053**	0.8 (0.7–1.0)	1.0 (0.8–1.2)	0.731
**Health region**					
Barcelona Lleida Tortosa Manresa	114/2389 (4.8) 198/5158 (3.8) 85/3419 (2.5) 123/3164 (3.9)	**<0.001**	reference 0.8 (0.6–1.0) 0.5 (0.4–0.7) 0.8 (0.6–1.0)	reference 1.0 (0.7–1.5) 0.7 (0.5–1.0) 1.0 (0.7–1.5)	**0.020**
**Visits during the intervention period**					
1 visit >1 visit	20/2193 (0.9) 500/11,937 (4.2)	**<0.001**	reference 4.7 (3.0–7.4)	reference 4.4 (2.8–6.8)	**<0.001**
**Time registered in the system**					
<5 years ≥5 years	170/3672 (4.6) 350/10,458 (3.4)	**<0.001**	reference 0.7 (0.6–0.9)	reference 0.8 (0.6–1.1)	0.150
**Comorbidity with an ID**^	34/482 (7.1)	**<0.001**	2.1 (1.4–2.9)	2.1 (1.5–3.1)	**<0.001**
**Intervention centres**				–	**–**
No Yes	304/7609 (4.0) 305/8171 (3.7)	0.392	reference 0.9 (0.8–1.1)		

+ Area of birth: Eastern Europe (EE), Latin-America (LA), Northern Africa (NA), and Sub-Saharan Africa (SSA); Infectious disease (ID).

^ Infectious diseases included in the study: Human immunodeficiency virus (HIV), Viral hepatitis B and C, Active Tuberculosis, Chagas disease, Strongyloidiasis and Schistosomiasis.

⁎ p-values were estimated using Chi-square test. **Note:** Simple and multiple logistic regressions were conducted to obtain odds ratio (OR), adjusted odds ratio (aOR) and 95 % CI of factors associated with mental disorders. The final model was adjusted by age, sex, area of birth, country of birth in conflict in 2017, health region, visits during the intervention period, time registered in the system and comorbidity with an ID.

### Type of mental health disorders

During the study period, 547 mental health diagnoses were made during the intervention period in 520 patients, of which 75.7 % (414/547) were new diagnoses. Precisely, out of 14,130 patients, 69 (0.5 %) were diagnosed with mood disorders, 346 (2.5 %) with anxiety disorders, and 127 (0.9 %) with sleeping disorders (Table 3). Being a woman was associated with the presence of mood disorders (51/7358; 0.7 %, *p* < 0.001) and anxiety disorders (240/7358; 3.3 %, *p* < 0.001). EEs (25/2971, 0.8 %, *p* = 0.004) presented mood disorders more often than the rest of the migrants, while anxiety disorders were most common among LAs (126/3483; 3.6 %, *p* < 0.001). Comorbidity with an infectious disease was associated with anxiety disorders (20/482; 4.2 %, *p* = 0.014) and sleeping disorders (9/482; 7.1 %, *p* = 0.030). Moreover, five cases were diagnosed with other mental health conditions, and these were also associated with presenting an infection (2/482; 0.4 %, *p* = 0.011) (Table 3).

**Table 3:** Distribution of total number of diagnostic categories (ICD-10).

Diagnostic groups (ICD-10)	Mood disorders (F30-F39)		Anxiety disorders* (F40-F48)		Sleeping disorders⁎⁎ (F50-F59 and G47)		Other disorders⁎⁎⁎	
	n/N (%)	*p*-value	n/N (%)	*p*-value	n/N (%)	*p*-value	n/N (%)	*p*-value
**Total**	69/14,130 (0.5)		346/14,130 (2.5)		127/14,130 (0.9)		5/14,130 (0.04)	
**Age category**								
<18 years 18–35 years 36–55 years >55 years	1/451 (0.2) 26/5241 (0.5) 32/7017 (0.5) 10/1421 (0.7)	0.591	8/451 (1.8) 140/5241 (2.7) 172/7017 (2.5) 26/1421 (1.8)	0.238	2/451 (0.4) 36/5241 (0.7) 74/7017 (1.1) 15/1421 (1.1)	0.118	1/451 (0.2) 1/5241 (0.02) 3/7017 (0.04) 0/1421 (0.0)	0.208
**Sex**								
Women Men	51/7358 (0.7) 18/6772 (0.3)	**<0.001**	240/7358 (3.3) 106/6772 (1.6)	**<0.001**	66/7358 (0.9) 61/6772 (0.9)	0.981	2/7358 (0.03) 3/6772 (0.04)	0.461
**Area of birth**+								
EE LA NA SSA Southern Asia and Middle East Eastern and Southeast Asia	25/2971 (0.8) 21/3483 (0.6) 18/4587 (0.4) 4/1789 (0.2) 0/886 (0.0) 1/414 (0.2)	**0.004**	93/2971 (3.1) 126/3483 (3.6) 89/4587 (1.9) 21/1789 (1.2) 11/886 (1.2) 6/414 (1.5)	**<0.001**	30/2971 (1.0) 37/3483 (1.1) 35/4587 (0.8) 18/1789 (1.0) 6/886 (0.7) 1/414 (0.2)	0.429	0/2971 (0.0) 1/3483 (0.03) 3/4587 (0.1) 1/1789 (0.1) 0/886 (0.0) 0/414 (0.0)	0.739
**Health region**								
Barcelona Lleida Tortosa Manresa	19/2389 (0.8) 22/5158 (0.4) 12/3419 (0.4) 16/3164 (0.5)	0.094	84/2389 (3.5) 117/5158 (2.3) 59/3419 (1.7) 86/3164 (2.7)	**<0.001**	18/2389 (0.8) 64/5158 (1.2) 19/3419 (0.6) 26/3164 (0.8)	**0.007**	0/2389 (0.0) 5/5158 (0.1) 0/3419 (0.0) 0/3164 (0.0)	**0.058**
**Comorbidity with an ID**^	5/482 (1.0)	0.086	20/482 (4.2)	**0.014**	9/482 (7.1)	**0.030**	2/482 (0.4)	**0.011**

+ Area of birth: Eastern Europe (EE), Latin-America (LA), Northern Africa (NA), and Sub-Saharan Africa (SSA); Infectious disease (ID).

^ Infectious diseases included in the study: Human immunodeficiency virus (HIV), Viral hepatitis B and C, Active Tuberculosis, Chagas disease, Strongyloidiasis and Schistosomiasis.

⁎ Anxiety, dissociative, stress-related, somatoform and other nonpsychotic disorders.

⁎⁎ Inorganic insomnia and sleep disorders.

⁎⁎⁎ Other disorders include mental and behavioural disorders due to psychoactive substance use (F10-F19), psychotic disorders (F20-F29), symptoms and signs involving emotional state (R45) and sleep disorder (G47). p-values were estimated using Fisher's exact or Chi-square tests.

Three patients (two who were women) were diagnosed with post-traumatic stress disorder (PTSD) (F43.1), of which only one recently arrived from a country in conflict, and two women were diagnosed with acute stress reaction (F43.0), both of whom were long residents in Catalonia and originated from non-conflicted countries. 40/14,130 (0.3 %) migrant patients were diagnosed with adjustment disorders (F43.2), mostly women (29/40, 72.5 %) who have been residing in Catalonia for ≥5 years (Annex 4). Of the five cases with other mental health conditions, two were diagnosed with symptoms and signs involving emotional state (R45) (e.g., nervousness), one with cocaine-related disorders (F14.0) and two with psychotic disorders (F23.0 and F23.2) (Annex 5). Annex 4 includes the detailed distribution of specific mental disorders in the cohort.

### Factors associated with mental health disorders

Women presented higher odds (aOR: 1.5, [95 % CI 1.2–1.8, *p* < 0.001]) of being diagnosed with a mental disorder than men when adjusting the model for age, sex, area of birth, countries in conflict in 2017, health region, visits during the intervention, time registered in the Catalan health system, and being diagnosed with an infectious disease (Table 2). In addition, migrants from SSA (aOR: 0.5, [95 %CI: 0.3–0.7]), NA (aOR: 0.7, [95 %CI 0.5–0.9]), Southern Asia and Middle East (aOR: 0.5, [95 %CI 0.3–0.8]), and Eastern and Southeast Asia (aOR: 0.3, [95 %CI 0.2–0.7]) were associated with lower odds of being diagnosed with a mental disorder when compared with migrant patients from EE countries (*p* < 0.001) (Table 2).

Mental disorders were most common in the PCCs located in Barcelona (114/2389; 4.5 %, *p* < 0.001) (Table 2), having higher odds of presenting a mental disorder compared with migrants attended at other health regions (*p* = 0.020) when adjusting the model (Table 2). Mental health disorders were also associated with having visited more than one time the PCCs during the intervention (500/11,937, 4.2 %; aOR: 4.4, [95 %CI 2.8–6.8, *p* < 0.001]) and with having an infection (34/482,7.1 %; aOR: 2.1, [95 %CI 1.5–3.1, *p* < 0.001]) (Table 2). Furthermore, even though no associations were found in the adjusted model, migrants coming from conflicted countries and individuals registered for ≥5years in the Catalan health system presented significantly lower odds of mental disorders compared with migrants coming from non-conflicted areas and those registered for <5 years, respectively (Table 2).

### Use of the CDSS

Healthcare professionals followed the screening strategy for only 29 of the 1840 (1.6 %) migrants coming from countries in conflict in 2017, attended at centres with the CDSS implemented; where 12 (41.4 %) of the questionnaires were addressed by professionals of the health region of Lleida and the other 17 (58.6 %) by professionals of the region of Manresa. Only one man of the 29 (3.4 %) migrant patients with an assessment performed was diagnosed with a mental disorder (Annex 3).

## Discussion

We have described a relatively low proportion (3.7 %) of migrant patients who were diagnosed with a mental disorder on a routine basis at primary care level. There are several explanations for this low proportion. Migrants are not likely to actively seek care for their mental health problems (Lu et al., 2020) mainly because of language barriers and unfamiliarity with the available healthcare services in the host country, cultural aspects of seeking help for mental health problems and coping strategies, as well as the stigma attached to mental health (Desa et al., 2020; Colucci et al., 2014).

Further, those migrants who reach primary care services may face barriers related to the cultural influences of the presentation and expression of their mental health symptoms as well as the lack of or inappropriate use of cultural mediators/interpreters (Kirmayer et al., 2003; Feldman, 2006). Migrants’ mental health needs are complex and multifaceted; for example, somatic symptoms can be the presentation of a mental disorder, which can also be part of the differential diagnosis of certain infectious diseases to which some migrant population groups are frequently exposed. This can result in undetected mental health problems or misdiagnoses of mental health conditions. Health professionals may be more focused on biomedical care, overlooking psychological distress, especially when faced with challenging medical conditions such as infectious diseases or physical ailments (Kroenke and Harris, 2001; Kroenke, 2003). Further, time limitations during medical visits may necessitate an almost exclusive focus on physical symptoms for patients with serious medical illnesses (Kroenke and Unutzer, 2017).

In our study's migrant population, anxiety disorders were the most common, followed by mood disorders and sleep disorders. Migrants usually live under highly distressing situations (unemployment, discrimination, and social exclusion) and overgoing uncertainties in their resettlement environments, which may have a negative impact on their mental health and wellbeing (Feyissa et al., 2022). Mental disorders were most common among women. Migrant women constitute a highly vulnerable group to mental disorders, especially anxiety and mood disorders. Particularly, for those migrants in the perinatal period, high rates of anxiety, prenatal depression (Iliadou et al., 2019) and postnatal depression (Schmied et al., 2017) have been reported. Women were also more likely to present PTSD and adjustment disorders, which may be a consequence of the physical violence experienced during the migration journey and psychosocial stressors in the host country. Our clinical results corroborate the findings from another qualitative study with general professionals, where they described that migrant women show a higher risk of mental health problems due to their difficulties in adapting to life in a new country, as a consequence of the migration process (Gonçalves et al., 2022).

In relation to the region of birth, Latin American migrants showed a higher proportion of mental disorders when compared to migrants from other regions. This finding may appear contrary to what should be expected given the notion of cultural congruity (Bhugra and Arya, 2005), which holds that cultural similarity correlates negatively with psychopathology. Additionally, despite the fact that age was not proved to be significant in relation with mental diagnosis, further research needs to be conducted towards this regard. Previous research highlights the special vulnerability of both accompanied and unaccompanied migrant minors (<18years) in terms of mental health (Kien et al., 2019).

Interestingly, mental health problems were more frequent among migrants registered in the primary healthcare centres in Barcelona city. Social stress is an important factor of mental disorders in cities, mostly related to social and economic disparities (e.g., income disparities, cost of living, incidence of crime) and environmental dimensions (e.g., air pollution, loss of green spaces) (Bhugra et al., 2019). Those who visited the health facilities more than once during the study period, and those migrants who were newly registered (<5 years) in the Catalan healthcare system, were more likely to present a mental disorder. Our study supports previous findings, which suggest that patients with medical unexplained physical symptoms (MUPS) and somatic representations of their mental health conditions are frequent visitors of health facilities (Vermeir et al., 2021). Further, high prevalence of mental disorders has been previously reported in newly arrived migrants in host countries (Serre-Delcor et al., 2021; Knights et al., 2022).

Having a concomitant infectious disease was also associated with mental health disorders, what have been previously reported in particular for those stigmatizing infections common in migrants such as HIV (Remien et al., 2019), Tuberculosis (Doherty et al., 2013) or Chagas diseases (Jackson et al., 2012)). On one side mental disorders are risk factors for the development of communicable (and non-communicable) diseases, since mental disorders may increase the transmission risk of certain infectious diseases (Deaterly et al., 2023; Matodzi et al., 2023). On the other side, some infectious diseases can increase the risk for mental disorders or exacerbate existing mental illness. In this regard, the subsequent comorbidity can complicate diagnosis, quality of care provided, treatment and adherence, and further affects treatment outcomes for both conditions (López et al., 2023; Prince et al., 2007).

Towards the improvement of screening and diagnosis for mental health problems in migrant patients at primary care, we tested a CDSS tool specific to migrant mental health. At the primary care centres where the tool was tested (intervention sites), the migrant mental health CDSS tool had not been proven useful towards the opportunistic screening of cases. Only 29 patients were screened; this suggests that mental health screening questionnaires implemented as part of the routine primary care visits may not be as feasible as screening for infectious diseases based on simply ordering laboratory tests (Sequeira-Aymar et al., 2021), due to the complexity of the questions to be addressed and the lack of time to address them properly. In addition, only in one screened patient a mental diagnosis was reported. Therefore, the screening criteria of the tool, -patients coming from countries in conflict in 2017-, may not be adequate for the detection of cases with mental health needs for two main reasons. The personal resilience factors may vary among migrants (Papadopoulos, 2021). Therefore, migrants’ response to adversity, such as displacement caused by war and conflict, is not limited to being traumatized but includes resilience in individual functioning and adaptation at different levels: individual, family, community and sociocultural (Papadopoulos, 2007). Secondly, the psychological impact of an armed conflict on an individual may differ depending on whether the person has been recently arrived from a country of armed conflict or s/he has been long time away from that context. This may be the reason why in our study, mental disorders were less frequent among long residing migrants coming from countries in conflict.

Based on the aforementioned reasons, the criterion for mental health screening as well as the items of the screening tool need to be revised considering the interplay of social determinants in mental health. For the purpose of the study, and both at intervention and control sites, a one-hour training was provided by a trained consultant psychiatrist specialized in migrant mental health. Health professionals at primary healthcare level could greatly benefit from follow-up trainings with a more comprehensive agenda regarding the mental health screening assessment of migrants.

### Limitations

Our results were limited to the analysis of data retrieved from routine data collection by health care professionals at primary health care. Therefore, sociodemographic characteristics of the migrant cohort had not been collected, other than age, sex, country of birth and time of registration at the national health system. Variables such as legal status, socioeconomic features, number of dependents, employment and time of stay in the country have not been included in the study. No distinction was thus made between refugees, asylum seekers, or undocumented migrants. The retrospective routine data collection may have led to inaccuracies or measurement errors since data were collected on a routine base without a specific epidemiological methodology. These data were extracted from the EHR system of the PCCs of the four health regions that were selected due to their high migration density (>20 %). Thus, the study findings can only be representative of the migrant population residing in these regions of Catalonia, Spain. An additional limitation is that the items of the mental health screening assessment tool had not been previously validated. Its statistical validation could have ensured the psychometric properties of the tool, its sensitivity and specificity dimensions.

We do acknowledge that there is a within-group variation that we are unable to account for. Migrants are not a homogeneous group (refugees vs immigrants) and the context (country of birth vs host country), as well as settings (clinical vs community) in which they are examined, influences the experience, expression and explanation of their mental health and psychosocial problems (Carta et al., 2005). Also, migrants born in the geographical areas of Northern and Southern Europe, Anglo-Saxon America, and Oceania were excluded from the analysis, which might have biased our results, because these countries were not considered in the definition of the target population for the mental health screening assessment supported by the CDSS.

The study entails some important clinical and policy implications. As the main point of access to mental health services for migrant patients, PCCs can play an important role in the early identification and treatment of mental disorders[18]. In 2008, the World Health Organization (WHO) and the World Organization of Family Doctors (WONCA) released a report to systematically promote the integration of mental health services into primary care (Collins, 2016), highlighting that best practices in screening and intervention services for mental disorders in migrants need to be sensitive to where individuals and their families are in the resettlement trajectory. The successful integration of mental health in primary care is highly dependent on the policy context. Our study has demonstrated important unmet mental health needs among migrants, as a result of social inequalities in health. Policy makers are thus informed on the urgency of improving the access and cultural relevance of mental healthcare. Strengthening capacity for migrant mental health in primary care can improve health equity outcomes by providing timely access to coordinated and integrated mental health care.

## Conclusions

The proportion of mental disorders was low among the migrant patients in our study, suggesting that mental health problems in migrants constitute a neglected condition resulting in mental health inequities at primary healthcare. Health policy actions need to consider sex, region of birth, settlement area at host country and frequency of medical visits as key factors for the experience, expression and manifestation of mental health conditions among migrants. Our screening assessment tool was not successfully applied and the screening criteria should be revised in order to account for the mental health needs of different migrant subgroups in primary care. Addressing the barriers that health professionals may experience in their clinical decision processes relies on public health investments that are integral, inclusive, and non-discriminatory.

## Data availability

Data available on request.

## CRediT authorship contribution statement

**Stella Evangelidou:** Conceptualization, Methodology, Data curation, Formal analysis, Writing – original draft, Writing – review & editing. **Angeline Cruz:** Conceptualization, Data curation, Formal analysis, Writing – original draft, Writing – review & editing. **Yolanda Osorio:** Funding acquisition, Conceptualization, Writing – review & editing. **Ethel Sequeira-Aymar:** Funding acquisition, Conceptualization, Project administration, Investigation, Writing – review & editing. **Alessandra Queiroga Gonçalves:** Conceptualization, Project administration, Investigation, Writing – review & editing. **Laura Camps-Vila:** Project administration, Investigation, Writing – review & editing. **Marta M. Monclús-González:** Project administration, Investigation, Writing – review & editing. **Alba Cuxart-Graell:** Writing – original draft, Writing – review & editing. **Elisa M. Revuelta-Muñoz:** Project administration, Investigation, Writing – review & editing. **Núria Busquet-Solé:** Project administration, Investigation, Writing – review & editing. **Susana Sarriegui-Domínguez:** Project administration, Investigation, Writing – review & editing. **Aina Casellas:** Methodology, Data curation, Formal analysis, Writing – review & editing. **M. Rosa Dalmau Llorca:** Project administration, Investigation, Writing – review & editing. **Carina Aguilar Martín:** Conceptualization, Project administration, Investigation, Writing – review & editing. **Ana Requena-Mendez:** Funding acquisition, Conceptualization, Methodology, Data curation, Formal analysis, Writing – original draft, Writing – review & editing.

## Declaration of Competing Interest

The authors declare that they have no known competing financial interests or personal relationships that could have appeared to influence the work reported in this paper.

## Appendix Supplementary materials

Image, application 1
