# Supplementary material for: Screening and routine diagnosis of mental disorders among migrants in primary care: A cross-sectional study
Source: J Migr Health. 2023 Nov 10;8:100205. doi: 10.1016/j.jmh.2023.100205 (PMC10692454; doi:10.1016/j.jmh.2023.100205)
Supplement: Supplementary file 1 [file mmc1.docx]

**Supplementary files**

**Annex 1. Classification of areas of birth**

| **Area of birth*** | **Sub-area of birth^** | **Countries** |
| --- | --- | --- |
| Eastern Europe (EE) | Eastern Europe | Armenia, Azerbaijan, Belarus, Czech Republic, Estonia, Russian Federation, Hungary, Kazakhstan, Kyrgyzstan, Latvia, Lithuania, Moldova, Poland, Romania, Serbia, Slovakia, Tajikistan, Ukraine People´s Republic, and Uzbekistan |
| Latin-America (LA) | Latin-America | Anguilla, Antigua and Barbuda, Netherlands Antilles, Argentina, Aruba, The Bahamas, Barbados, Belize, Bermuda Islands, Bolivia, Bosnia and Herzegovina, Brazil, Cayman Islands, Chile, Colombia, Costa Rica, Cuba, Dominica, Dominican Republic, Ecuador, El Salvador, Falkland Islands, French Guiana, Grenada, Guadeloupe, Guatemala, Guyana, Haiti, Honduras, Jamaica, Martinique, Mexico, Montserrat, Nicaragua, Panama, Paraguay, Peru, Puerto Rico, Saint Christopher and Nevis, Saint Lucia, Saint Vincent and the Grenadines, South Georgia and the South Sandwich Islands, Surinam, Trinidad and Tobago, Turks and Caicos Islands, Uruguay, Venezuela, Virgin Islands (USA), and Virgin Islands (Great Britain) |
| Northern Africa (NA) | Northern Africa | Algeria, Egypt, Libya, Morocco, Tunisia, and Western Sahara |
| Sub-Saharan Africa (SSA) | Sub-Saharan Africa | Angola, Botswana, Benin, Burkina Faso, Burundi, Cameroon, Cape Verde, Central African Republic, Chad, Comoros, Congo, Democratic Republic of Congo, Djibouti, Equatorial Guinea, Eritrea, Ethiopia, Gabon, Gambia, Ghana, Guinea, Guinea Bissau, Ivory Coast, Kenya, Lesotho, Liberia, Madagascar, Malawi, Mali, Mauritia, Mauritania, Mayotte, Mozambique, Namibia, Niger, Nigeria, Republic of South Africa, Réunion, Rwanda, Saint Helena, Saint Thomas and Prince, Senegal, Seychelles, Sierra Leone, Somalia, Sudan, Swaziland, United Republic of French Tanzania, Togo, Uganda, Zambia, and Zimbabwe |
| Southern Asia and Middle East | Southern Asia and Middle East | Afghanistan, Saudi Arabia, Bahrain, India, Iraq, Islamic Republic of Iran, Israel, Jordan, Kuwait, Lebanon, Oman, Pakistan, Qatar, Syria, Territory Occupied by Palestine, Turkmenistan, United Arab Emirates, and Yemen |
| Eastern and Southeast Asia | Eastern and Southeast Asia | Bangladesh, Bhutan, Brunei, Cambodia, China, Republic of Korea, Democratic People’s Republic of Korea, Hong Kong, Indonesia, Japan, Philippines, Lao People’s Democratic Republic, Macau, Malaysia, Mongolia, Myanmar, Nepal, Singapore, Sri Lanka, Thailand, Taiwan, East Timor, and Vietnam |
| Western countries^+^ | Northern Europe | Aland Islands, Austria, Belgium, Bouvet Island (Norway), Denmark, Faroe Islands, Finland, Georgia, Germany, Guernsey, Iceland, Ireland, Isle of Man, Jersey, Liechtenstein, Luxembourg, Monaco, Norway, Netherlands, United Kingdom, Sweden, Switzerland, Svalbard and Jan Mayen, and French Southern and Antarctic Land |
|  | Southern Europe | Albania, Andorra, Bulgaria, Croatia, Cyprus, Slovenia, France, Gibraltar, Greece, Italy, North Macedonia, Malta, Montenegro, Portugal, San Marino, Turkey, and Vatican City |
|  | Anglo-Saxon America | Canada, United States of America, United States Minor Outlying Islands, and Saint-Pierre and Miquelon |
|  | Oceania | Australia, British Indian Ocean Territory, Christmas Island, Cocos Islands, Cook Islands, Federated States of Micronesia, Fiji, French Polynesia, Guam, Heard and McDonald Islands, Kiribati, Maldives, Northern Mariana Islands, Marshall Islands, Nauru, Niue, Norfolk Island, New Caledonia, New Zealand, Palau, Papa New Guinea, Pitcairn, Solomon, Samoa, American Samoa, Tokelau, Tonga, Tuvalu, Vanuatu, and Wallis and Futuna |
| *Classification used for the analysis; ^Inspired by the GeoSentinel classification but has been adapted by Fundació Institut Universitari per a la recerca a l'Atenció Primària de Salut Jordi Gol i Gurina the Jordi Gol Primary Care Research Institute (IDIAPJGol); ^+^Areas excluded from the study. | | |

**Annex 2. STROBE Guidelines Checklist**

|  | **Item No** | **Recommendation** | **Page No** |
| --- | --- | --- | --- |
| **Title and abstract** | 1 | (*a*) Indicate the study’s design with a commonly used term in the title or the abstract | 1 |
|  |  | (*b*) Provide in the abstract an informative and balanced summary of what was done and what was found | 3,4 |
| **Introduction** | | |  |
| Background/rationale | 2 | Explain the scientific background and rationale for the investigation being reported | 5,6 |
| Objectives | 3 | State specific objectives, including any prespecified hypotheses | 6 |
| **Methods** | | |  |
| Study design | 4 | Present key elements of study design early in the paper | 6,7 |
| Setting | 5 | Describe the setting, locations, and relevant dates, including periods of recruitment, exposure, follow-up, and data collection | 6-8 |
| Participants | 6 | (*a*) Give the eligibility criteria, and the sources and methods of selection of participants. Describe methods of follow-up | 7 |
|  |  | (*b*) For matched studies, give matching criteria and number of exposed and unexposed | N/A |
| Variables | 7 | Clearly define all outcomes, exposures, predictors, potential confounders, and effect modifiers. Give diagnostic criteria, if applicable | 8,9 |
| Data sources/ measurement | 8* | For each variable of interest, give sources of data and details of methods of assessment (measurement). Describe comparability of assessment methods if there is more than one group | 8,9 |
| Bias | 9 | Describe any efforts to address potential sources of bias | 6,7 |
| Study size | 10 | Explain how the study size was arrived at | N/A |
| Quantitative variables | 11 | Explain how quantitative variables were handled in the analyses. If applicable, describe which groupings were chosen and why | 8,9 |
| Statistical methods | 12 | (*a*) Describe all statistical methods, including those used to control for confounding | 9 |
|  |  | (*b*) Describe any methods used to examine subgroups and interactions | 9 |
|  |  | (*c*) Explain how missing data were addressed | 9 |
|  |  | (*d*) If applicable, explain how loss to follow-up was addressed | N/A |
|  |  | (*e*) Describe any sensitivity analyses | N/A |
| **Results** | | |  |
| Participants | 13* | (a) Report numbers of individuals at each stage of study—eg numbers potentially eligible, examined for eligibility, confirmed eligible, included in the study, completing follow-up, and analysed | 10, Figure 1 |
|  |  | (b) Give reasons for non-participation at each stage | 10, Figure 1 |
|  |  | (c) Consider use of a flow diagram | Figure 1 |
| Descriptive data | 14* | (a) Give characteristics of study participants (eg demographic, clinical, social) and information on exposures and potential confounders | 10, Table 1 |
|  |  | (b) Indicate number of participants with missing data for each variable of interest | N/A |
|  |  | (c) Summarise follow-up time (eg, average and total amount) | N/A |
| Outcome data | 15* | Report numbers of outcome events or summary measures over time | 10,11, Table 1 and 2 |
| Main results | 16 | (*a*) Give unadjusted estimates and, if applicable, confounder-adjusted estimates and their precision (eg, 95% confidence interval). Make clear which confounders were adjusted for and why they were included | 12, Table 2 |
|  |  | (*b*) Report category boundaries when continuous variables were categorized | 10-12, Table 1-3 |
|  |  | (*c*) If relevant, consider translating estimates of relative risk into absolute risk for a meaningful time period | N/A |
| Other analyses | 17 | Report other analyses done—eg analyses of subgroups and interactions, and sensitivity analyses | 11,12, Table 3,  Annex 3, Annex 4 |
| **Discussion** | | |  |
| Key results | 18 | Summarise key results with reference to study objectives | 13 |
| Limitations | 19 | Discuss limitations of the study, taking into account sources of potential bias or imprecision. Discuss both direction and magnitude of any potential bias | 16,17 |
| Interpretation | 20 | Give a cautious overall interpretation of results considering objectives, limitations, multiplicity of analyses, results from similar studies, and other relevant evidence | 13-16 |
| Generalisability | 21 | Discuss the generalisability (external validity) of the study results | 17 |
| **Other information** | | |  |
| Funding | 22 | Give the source of funding and the role of the funders for the present study and, if applicable, for the original study on which the present article is based | 18 |

**Annex 3. Description of the migrant patients screened for mental disorders**

|  | **Barcelona** | **Lleida** | **Tortosa** | **Manresa** | **Total** |
| --- | --- | --- | --- | --- | --- |
|  | **n/N (%)** | **n/N (%)** | **n/N (%)** | **n/N (%)** | **n/N (%)** |
| Total | 0 | 12 | 0 | 17 | 29 |
| **Age category**  <18 years  18-35 years  36-55 years  >55 years | -  -  -  - | -  3/12 (25.0)  8/12 (66.7)  1/12 (8.3) | -  -  -  - | -  8/17 (47.0)  9/17 (52.9)  0/17 (0.0) | -  11/29 (37.9)  17/29 (58.6)  1/29 (3.5) |
| **Sex**  Women  Men | -  - | 2/12 (16.7)  10/12 (83.3) | -  - | 11/17 (64.7)  6/17 (35.3) | 13 (44.8)  16 (55.2) |
| **Area of birth^+^**  EE  LA  NA  SSA  Southern Asia and Middle East  Eastern and Southeast Asia | -  -  -  -  -  - | 1/12 (8.3)  3/12 (25.0)  3/12 (25.0)  3/12 (25.0)  2/12 (16.7)  0/12 (0.0) | -  -  -  -  -  - | 0/17 (0.0)  4 /17(23.5)  8/17 (47.1)  3/17 (17.7)  2/17 (11.8)  0/12 (0.0) | 1 (3.5)  7 (24.1)  11 (37.9)  6 (20.7)  4 (13.8)  - |
| **Year of arrival*** | - | 2004 (1999-2010) | - | 2008 (2003-2014) | 2005 (2002-2012) |
| **Episode of violence during migration journey** | - | 1/12 (8.3) | - | 0/7 (0.0) | 1/19 (5.3) |
| **Substance abuse** | - | 1/11 (9.1) | - | 1/6 (16.7) | 2/17 (11.8) |
| **Adjustment disorders** | - | 1/12 (8.3) | - | 3/7 (42.9) | 4/19 (21.1) |
| **Sleeping disorders** | - | 2/12 (16.7) | - | 4/7 (57.1) | 6/19 (31.6) |
| **Difficulty falling asleep** | - | 0/12 (0.0) | - | 3/7 (42.9) | 3/19 (15.8) |
| **Disruptive sleep disorders** | - | 4/12 (33.3) | - | 4/7 (57.1) | 8/19 (42.1) |
| **Insomnia** | - | 3/11 (27.3) | - | 4/7 (57.1) | 7/18 (38.9) |
| **Individuals diagnosed with at least one mental disorder** | - | 1/12 (8.3) | - | 0/17 (0.0) | 1/29 (3.5) |
| ^+^See Annex 1 for details on the classification of countries in areas of birth: Eastern Europe (EE), Latin-America (LA), Northern Africa (NA), and Sub-Saharan Africa (SSA); Infectious diseases (ID); ^Infectious diseases included in the study: Human immunodeficiency virus (HIV), Viral hepatitis B and C, Active Tuberculosis, Chagas disease, Strongyloidiasis and Schistosomiasis; *Presented in median and IQR | | | | | |

**Annex 4. Distribution of mental disorders (ICD-10)**

| **Diagnostic code (ICD-10)** | **Number of diagnosis (%)** | **Sex (percentage %)** | **Age category (percentage %)** | **Area of birth (percentage %)** | **Health region (percentage %)** |
| --- | --- | --- | --- | --- | --- |
| **Cocaine related disorders (F14.0)** | 1/14,130 (0.0) | M: 1/6,772 (0.0) | 36-55: 1/7,017 (0.0) | NA: 1/4,587 (0.0) | Lleida: 1/5,158 (0.0) |
| **Brief psychotic disorder (F23.0)** | 1/14,130 (0.0) | M: 1/6,772 (0.0) | 36-55: 1/7,017 (0.0) | SSA: 1/1,789 (0.0) | Lleida: 1/5,158 (0.0) |
| **Acute psychotic disorder (F23.2)** | 1/14,130 (0.0) | W: 1/7,358 (0.0) | 18-35: 1/5,241 (0.0) | LA: 1/3,483 (0.0) | Lleida: 1/5,158 (0.0) |
| **MOOD AFFECTIVE DISORDERS (F30-F39)** | | | | | |
| **Bipolar affective disorder, unspecified (F31.9)** | 5/14,130 (0.0) | W: 3/7,358 (0.0)  M: 2/6,772 (0.0) | 18-35: 3/5,241 (0.1)  36-55: 1/7,017 (0.0)  >55: 1/1,421 (0.1) | EE: 3/2,971 (0.1)  LA: 1/3,483 (0.0)  NA: 1/4,587 (0.0) | Barcelona: 2/2,389 (0.1)  Lleida: 1/5,158 (0.0)  Manresa: 2/3,164 (0.1) |
| **Depressive episodes (F32)** | 55/14,130 (0.4) | W: 40/7,358 (0.5)  M: 15/6,772 (0.2) | <18: 1/451 (0.2)  18-35: 21/5,241 (0.4)  36-55: 29/7,017 (0.4)  >55: 4/1,421 (0.3) | EE: 18/2,971 (0.6)  LA: 17/3,483 (0.5)  NA: 16/4,587 (0.4)  SSA: 4/1,789 (0.2) | Barcelona: 16/2,389 (0.7)  Lleida: 17/5,158 (0.3)  Tortosa: 9/3,419 (0.3)  Manresa: 13/3,164 (0.4) |
| **Major depressive disorder, single episode, mild (F32.0)** | 2/14,130 (0.0) | W: 2/7,358 (0.0) | 36-55: 2/7,017 (0.0) | LA: 2/3,483 (0.1) | Lleida: 1/5,158 (0.0)  Manresa: 1/3,164 (0.0) |
| **Major depressive disorder, single episode, moderate (F32.1)** | 1/14,130 (0.0) | W: 1/7,358 (0.0) | 36-55: 1/7,017 (0.0) | LA: 1/3,483 (0.0) | Tortosa: 1/3,419 (0.0) |
| **Major depressive disorder, single episode, severe without psychotic features (F32.2)** | 7/14,130 (0.1) | W: 5/7,358 (0.1)  M: 2/6,772 (0.0) | 18-35: 4/5,241 (0.1)  36-55: 3/7,017 (0.0) | EE: 3/2,971 (0.1)  LA: 2/3,483 (0.1)  NA: 1/4,587 (0.0)  SSA: 1/1,789 (0.1) | Barcelona: 2/2,389 (0.1)  Lleida: 3/5,158 (0.1)  Tortosa: 2/3,419 (0.1) |
| **Major depressive disorder, single episode, unspecified (F32.9)** | 45/14,130 (0.3) | W: 32/7,358 (0.4)  M: 13/6,772 (0.2) | <18: 1/451 (0.2)  18-35: 17/5,241 (0.3)  36-55: 23/7,017 (0.3)  >55: 4/1,421 (0.3) | EE: 15/2,971 (0.5)  LA: 12/3,483 (0.3)  NA: 15/4,587 (0.3)  SSA: 3/1,789 (0.2) | Barcelona: 14/2,389 (0.6)  Lleida: 13/5,158 (0.3)  Tortosa: 6/3,419 (0.2)  Manresa: 12/3,164 (0.4) |
| **Major depressive disorder, recurrent (F33)** | 3/14,130 (0.0) | W: 2/7,358 (0.0)  M: 1/6,772 (0.0) | >55: 3/1,421 (0.2) | EE: 2/2,971 (0.1)  NA: 1/4,587 (0.0) | Lleida: 1/5,158 (0.0)  Tortosa: 2/3,419 (0.1) |
| **Major depressive disorder, recurrent, mild (F33.0)** | 2/14,130 (0.0) | W: 1/7,358 (0.0)  M: 1/6,772 (0.0) | >55: 2/1,421 (0.1) | EE: 1/2,971 (0.0)  NA: 1/4,587 (0.0) | Lleida: 1/5,158 (0.0)  Tortosa: 1/3,419 (0.0) |
| **Major depressive disorder, recurrent, severe with psychotic symptoms (F33.3)** | 1/14,130 (0.0) | W: 1/7,358 (0.0) | >55: 1/1,421 (0.1) | EE: 1/2,971 (0.0) | Tortosa: 1/3,419 (0.0) |
| **Dysthymic disorder (F34.1)** | 7/14,130 (0.1) | W: 7/7,358 (0.1) | 18-35: 2/5,241 (0.0)  36-55: 3/7,017 (0.0)  >55: 2/1,421 (0.1) | EE: 3/2,971 (0.1)  LA: 3/3,483 (0.1)  Eastern and Southeast Asia: 1/414 (0.2) | Barcelona: 1/2,389 (0.0)  Lleida: 4/5,158 (0.1)  Tortosa: 1/3,419 (0.0)  Manresa: 1/3,164 (0.0) |
| **ANXIETY, DISSOCIATIVE, STRESS-RELATED, SOMATOFORM AND OTHER NONPSYCHOTIC MENTAL DISORDERS (F40-F48)** | | | | | |
| **Phobic anxiety disorders (F40)** | 8/14,130 (0.1) | W: 6/7,358 (0.1)  M: 2/6,772 (0.0) | <18: 1/451 (0.2)  18-35: 3/5,241 (0.1)  36-55: 4/7,017 (0.1) | EE: 1/2,971 (0.0)  LA: 3/3,483 (0.1)  NA: 3/4,587 (0.1)  Eastern and Southeast Asia: 1/414 (0.2) | Barcelona: 5/2,389 (0.2)  Lleida: 1/5,158 (0.0)  Manresa: 2/3,164 (0.1) |
| **Specific (isolated) phobias (F40.2)** | 6/14,130 (0.0) | W: 5/7,358 (0.1)  M: 1/6,772 (0.0) | 18-35: 3/5,241 (0.1)  36-55: 3/7,017 (0.0) | EE: 1/2,971 (0.0)  LA: 2/3,483 (0.1)  NA: 2/4,587 (0.0)  Eastern and Southeast Asia: 1/414 (0.2) | Barcelona: 4/2,389 (0.2)  Manresa: 2/3,164 (0.1) |
| **Phobic anxiety disorder, unspecified (F40.9)** | 2/14,130 (0.0) | W: 1/7,358 (0.0)  M: 1/6,772 (0.0) | <18: 1/451 (0.2)  36-55: 1/7,017 (0.0) | LA: 1/3,483 (0.0)  NA: 1/4,587 (0.0) | Barcelona: 1/2,389 (0.0)  Lleida: 1/5,158 (0.0) |
| **Other anxiety disorders (F41)** | 298/14,130 (2.1) | W: 205/7,358 (2.3)  M: 93/6,772 (1.4) | <18: 5/451 (1.1)  18-35: 122/5,241 (2.3)  36-55: 147/7,017 (2.1)  >55: 24/1,421 (1.7) | EE: 81/2,971 (2.7)  LA: 107/3,483 (3.1)  NA: 76/4,587 (1.7)  SSA: 20/1,789 (1.1)  Southern Asia and Middle East: 9/886 (1.0)  Eastern and Southeast Asia: 5/414 (1.2) | Barcelona: 74/2,389 (3.1)  Lleida: 101/5,158 (2.0)  Tortosa: 47/3,419 (1.4)  Manresa: 76/3,164 (2.4) |
| **Panic disorder (episodic paroxysmal anxiety) (F41.0)** | 3/14,130 (0.0) | W: 3/7,358 (0.0) | 18-35: 2/5,241 (0.0)  36-55: 1/7,017 (0.0) | EE: 2/2,971 (0.1)  NA: 1/4,587 (0.0) | Lleida: 2/5,158 (0.0)  Tortosa: 1/3,419 (0.0) |
| **Generalized anxiety disorder (F41.1)** | 8/14,130 (0.1) | W: 7/7,358 (0.1)  M: 1/6,772 (0.0) | <18: 1/451 (0.2)  18-35: 4/5,241 (0.1)  36-55: 3/7,017 (0.0) | EE: 1/2,971 (0.0)  LA: 3/3,483 (0.1)  NA: 4/4,587 (0.1) | Barcelona: 3/2,389 (0.1)  Lleida: 1/5,158 (0.0)  Tortosa: 1/3,419 (0.0)  Manresa: 3/3,164 (0.1) |
| **Mixed anxiety and depressive disorder (F41.2)** | 14/14,130 (0.1) | W: 8/7,358 (0.1)  M: 6/6,772 (0.1) | 18-35: 6/5,241 (0.1)  36-55: 7/7,017 (0.1)  >55: 1/1,421 (0.1) | LA: 5/3,483 (0.1)  NA: 8/4,587 (0.2)  Southern Asia and Middle East: 1/886 (0.1) | Barcelona: 2/2,389 (0.1)  Lleida: 7/5,158 (0.1)  Manresa: 5/3,164 (0.2) |
| **Other mixed anxiety disorders (F41.3)** | 2/14,130 (0.0) | W: 2/7,358 (0.0) | 18-35: 1/5,241 (0.0)  36-55: 1/7,017 (0.0) | LA: 1/3,483 (0.0)  NA: 1/4,587 (0.0) | Barcelona: 2/2,389 (0.1) |
| **Anxiety disorder, unspecified (F41.9)** | 277/14,130 (2.0) | W: 189/7,358 (2.6)  M: 88/6,772 (1.3) | <18: 4/451 (0.9)  18-35: 115/5,241 (2.2)  36-55: 135/7,017 (1.9)  >55: 23/1,421 (1.6) | EE: 79/2,971 (2.7)  LA: 98/3,483 (2.8)  NA: 67/4,587 (1.5)  SSA: 20/1,789 (1.1)  Southern Asia and Middle East: 8/886 (0.9)  Eastern and Southeast Asia: 5/414 (1.2) | Barcelona: 69/2,389 (2.9)  Lleida: 92/5,158 (1.8)  Tortosa: 46/3,419 (1.4)  Manresa: 70/3,164 (2.2) |
| **Obsessive-compulsive disorder, unspecified (F42.9)** | 1/14,130 (0.0) | M: 1/6,772 (0.0) | 18-35: 1/5,241 (0.0) | NA: 1/4,587 (0.0) | Lleida: 1/5,158 (0.0) |
| **Acute stress reaction (F43.0)** | 2/14,130 (0.0) | W: 2/7,358 (0.0) | 36-55: 2/7,017 (0.0) | EE: 1/2,971 (0.0)  NA: 1/4,587 (0.0) | Lleida: 1/5,158 (0.0)  Tortosa: 1/3,419 (0.0) |
| **Post-traumatic stress disorder (PTSD) (F43.1)** | 3/14,130 (0.0) | W: 2/7,358 (0.0)  M: 1/6,772 (0.0) | <18: 1/451 (0.2)  18-35: 1/5,241 (0.0)  36-55: 1/7,017 (0.0) | EE: 1/2,971 (0.0)  NA: 2/4,587 (0.0) | Lleida: 2/5,158 (0.0)  Tortosa: 1/3,419 (0.0) |
| **Adjustment disorders (F43.2)** | 40/14,130 (0.3) | W: 29/7,358 (0.4)  M: 11/6,772 (0.2) | <18: 2/451 (0.4)  18-35: 15/5,241 (0.3)  36-55: 21/7,017 (0.3)  >55: 2/1,421 (0.1) | EE: 12/2,971 (0.4)  LA: 17/3,483 (0.5)  NA: 9/4,587 (0.2)  Southern Asia and Middle East: 2/886 (0.2) | Barcelona: 5/2,389 (0.2)  Lleida: 13/5,158 (0.3)  Tortosa: 12/3,419 (0.4)  Manresa: 10/3,164 (0.3) |
| **Dissociative fugue (F44.1)** | 2/14,130 (0.0) | W: 1/7,358 (0.0)  M: 1/6,772 (0.0) | 18-35: 2/5, 241 (0.0) | SSA: 2/1,789 (0.1) | Lleida: 2/5,158 (0.0) |
| **BEHAVIOURAL SYNDROMES ASSOCIATED WITH PHYSIOLOGICAL FACTORS (F50-F59)** | | | | | |
| **Nonorganic insomnia (F51.0)** | 118/14,130 (0.8) | W: 60/7,358 (0.8)  M: 58/6,772 (0.9) | <18: 1/451 (0.2)  18-35: 35/5,241 (0.7)  36-55: 68/7,017 (1.0)  >55: 14/1,421 (1.0) | EE: 27/2,971 (0.9)  LA: 33/3,483 (1.0)  NA: 33/4,587 (0.7)  SSA: 18/1,789 (1.0)  Southern Asia and Middle East: 6/886 (0.7)  Eastern and Southeast Asia: 1/414 (0.2) | Barcelona: 16/2,389 (0.7)  Lleida: 61/5,158 (1.2)  Tortosa: 18/3,419 (0.5)  Manresa: 23/3,164 (0.7) |
| **Sleep disorders (G47)** | 10/14,130 (0.1) | W: 7/7,358 (0.1)  M: 3/6,772 (0.0) | <18: 1/451 (0.2)  18-35: 1/5,241 (0.0)  36-55: 6/7,017 (0.1)  >55: 2/1,421 (0.1) | EE: 3/2,971 (0.1)  LA: 5/3,483 (0.1)  NA: 2/4,587 (0.0) | Barcelona: 2/2,389 (0.1)  Lleida: 4/5,158 (0.1)  Tortosa: 1/3,419 (0.0)  Manresa: 3/3,164 (0.1) |
| **Symptoms and signs involving emotional state (R45)** | 2/14,130 (0.0) | W: 1/7,358 (0.0)  M: 1/6,772 (0.0) | <18: 1/451 (0.2)  36-55: 1/7,017 (0.0) | NA: 2/4,587 (0.0) | Lleida: 2/5,158 (0.1) |
| Sex: W=Women, and M=Men; Age category: <18 years, 18-35 years, 36-55 years, and >55 years; Area of birth: Eastern Europe (EE), Latin-America (LA), Northern Africa (NA), Sub-Saharan Africa (SSA), and European Union, Anglo-Saxon America and Oceania (EU-ASA & Oceania); Health region: Barcelona, Lleida, Tortosa, and Lleida | | | | | |
